# Comparison for immunophysiological responses of Jeju and Thoroughbred horses after exercise

**DOI:** 10.5713/ajas.19.0260

**Published:** 2019-08-03

**Authors:** Saichit Khummuang, Hyo Gun Lee, Sang Seok Joo, Jeong-Woong Park, Jae-Young Choi, Jin Hyeog Oh, Kyoung Hwan Kim, Hyun-Hee Youn, Myunghoo Kim, Byung-Wook Cho

**Affiliations:** 1 Department of Animal Science, College of Natural Resources and Life Sciences, Pusan National University, Miryang 50463, Korea

**Keywords:** Jeju Horse, Thoroughbred Horse, Exercise, Blood Analysis, Immunological Gene Expression

## Abstract

**Objective:**

The study was conducted to investigate variations in the immunophysiological responses to exercise-induced stress in Jeju and Thoroughbred horses.

**Methods:**

Blood samples were collected from the jugular veins of adult Jeju (n = 5) and Thoroughbred (n = 5) horses before and after 30 min of exercise. The hematological, biochemical, and immunological profiles of the blood samples were analyzed. Blood smears were stained and observed under a microscope. The concentration of cell-free (cf) DNA in the plasma was determined using real time polymerase chain reaction (PCR). Peripheral blood mononuclear cells (PBMCs) and polymorphonuclear cells were separated using Polymorphprep, and the expression of various stress-related and chemokine receptor genes was measured using reverse transcriptase (RT) and real-time PCR.

**Results:**

After exercise, Jeju and Thoroughbred horses displayed stress responses with significantly increased rectal temperatures, cortisol levels, and muscle catabolism-associated metabolites. Red blood cell indices were significantly higher in Thoroughbred horses than in Jeju horses after exercise. In addition, exercise-induced stress triggered the formation of neutrophil extracellular traps (NETs) and reduced platelet counts in Jeju horses but not in Thoroughbred horses. Heat shock protein 72 and heat shock protein family A (Hsp70) member 6 expression is rapidly modulated in response to exercise-induced stress in the PBMCs of Jeju horses. The expression of CXC chemokine receptor 4 in PBMCs was higher in Thoroughbred horses than in Jeju horses after exercise.

**Conclusion:**

In summary, the different immunophysiological responses of Jeju and Thoroughbred horses explain the differences in the physiological and anatomical properties of the two breeds. The physiology of Thoroughbred horses makes them suitable for racing as they are less sensitive to exercise-induced stress compared to that of Jeju horses. This study provides a basis for investigating the link between exercise-induced stresses and the physiological alteration of horses. Hence, our findings show that some of assessed parameters could be used to determine the endurance performance of horses.

## INTRODUCTION

Horses (*Equus caballus*) have been domesticated by humans worldwide for over 6,000 years. Today, horses are mainly used in horse racing or in equestrian sports. Historically, Thoroughbred horses were selected for human domestication and use based on their agility, stamina, and speed. In Korea, Thoroughbred horses are mainly used in the horse industry; however, the Jeju horse is becoming more popular in this regard because of its uniqueness, availability, and importance in Jeju Island. The Jeju horse is a species native to Korea (Natural Monument number 347), having remained isolated from the mainland for a large amount of time. Thus, the species has become well-adapted to the specific environmental conditions and selection pressures of Jeju island. Compared to Thoroughbred horses, Jeju horses are short, slow, hardy, and have strong immune systems, likely owing to their highly enriched immune-related nonsynonymous genes [1]. Therefore, the identification of Jeju horse-specific traits via comparative study would aid in the utilization and development of their useful characteristics [2,3].

Thoroughbred horse is the best breed for racing perfor mance [4]. Thoroughbred horses have been intensively studied as model organisms in the study of exercise, especially at the molecular level, with the Thoroughbred horse genome being well-studied using RNA-seq and the associated epigenetics also receiving significant attention from academics [5,6]. Comparative studies have investigated the differences between Thoroughbred and Jeju horses in order to identify genetic differences using microsatellites [7] and single nucleotide polymorphism (SNP) chip arrays [8,9]. In Jeju horses, nonsynonymous SNPs are overrepresented in immune response genes including toll-like receptor genes [1]. Physical activity has numerous effects on a variety of metabolic processes in the body, including thermodynamic and physiological biochemical processes (the activity of enzymes, hormones, chemokines, and cytokines). For example, exercise induced-hypoxia results in coordinate changes in the expression of various genes that are responsible for the induction of associated physiological changes [9–11]. Although many studies have investigated the physiological responses of Thoroughbred horses to exercise [12], few have investigated changes in the physiological characteristics of Jeju horses in response to exercise.

The hematological and biochemical parameters of the blood provide important information about the health status and metabolism of animals, and it is often more practical to perform hematological assessments than muscle biopsies, which are comparatively more complex and introduce risks associated with animal anesthetization. Therefore, this study aimed to identify novel traits that could be used in breeding programs to improve the physical performance of Jeju horses and reduce associated exercise-related stresses.

## MATERIALS AND METHODS

### Animals

Ten healthy horses (average age: 3.5 years old): five Jeju horses (Jeju group; n = 5) and five Thoroughbred horses (Thoroughbred group; n = 5) were used in the study. Heights and weights of Jeju horses were 115 to 125 cm and 244 to 249 kg, respectively. Whereas, heights and body weights of Thoroughbred horses were 150 to 173 cm and 450 to 500 kg, respectively. For health status of every horse was monitored for a week until the start date of experiment. Horses were observed without signs of injury, illness and medical records including; vital signs (heart rate, temperature, and respiration rate), ear, eye, nose, skin, coat, feet, limb, appetite, and attitude (bright and alert). Guidelines of horse managements were in compliance with international standards and “Korea Racing Authority” (http://www.kra.co.kr). All procedures used in the experiment were approved by the Pusan National University-Institutional Animal Care and Use Committee (Approval Number: PNU-2015-0864).

### Physical exercise and sample collection

Horses were exercised (longeing; circle diameter 11 m) for 30 min without rest. Before and after exercise, rectal temperature of each horse was measured by thermometer. Heart rate was measured by stethoscope in beats per minute. Blood samples (approximately 20 mL) were collected from the jugular vein. Samples were then separated using BD Vacutainer spray-coated K_2_ ethylenediaminetetraacetic acid (EDTA) Tubes for hematological analysis. BD Vacutainer SST Tubes contain spray-coated silica and a polymer gel were used to facilitate serum biochemical and immunological analysis. BD Vacutainer Citrate Tubes and a 3.2% buffered sodium citrate solution were used in routine fibrinogen analyses. For each blood sample, the following biochemical parameters were assessed: cortisol level; activities of aspartate aminotransferase, alanine aminotransferase, lactate dehydrogenase, creatine kinase, and alkaline phosphatase; total bilirubin; blood urea nitrogen (BUN); and creatinine (Cr) levels. Hematological parameters, including red blood cell (RBC) count, hemoglobin (Hb), hematocrit (Hct), mean cell (or corpuscular) volume (MCV), mean cell hemoglobin (MCH), mean cell hemoglobin concentration (MCHC), white blood cell (WBC) count, proportion of blood immune cells (percentage of lymphocytes, neutrophils, monocytes, eosinophils, and basophils), platelet count, fibrinogen level and immunological parameters, including immunoglobulin (Ig) G and IgM, were measured using an Auto Analyzer (Seegene, Seoul, Korea). The BUN/Cr ratio was also calculated to assess muscle catabolism.

### Blood smears and staining

One drop of blood from each sample was placed on a glass slide and gently spread using the edge of another glass slide. Blood smears were then air-dried, fixed in methanol, and stained according to the standard use of Leishman-Giemsa stain (Sigma-Aldrich, St. Louis, MO, USA).

### Quantification of cell free DNA

Plasma samples were centrifuged at 4°C at 16,000×*g* for 5 min to remove cell debris. The supernatant was diluted in sterile nuclease-free water at a 1:40 ratio for direct quantitative polymerase chain reaction (qPCR) measurement. The diluted plasma samples were stored at −20°C until they were analyzed. The cell free DNA (cfDNA) concentrations of the plasma were quantified using direct qPCR and a primer set (Table 1), producing an 88-bp fragment of the chromosomal myostatin (MSTN) amplicon [13]. Acting as a template, the diluted plasma was added to master mixer 20X Evagreen (SolGent, Seoul, Korea) containing 10 pmol primer set, 25 mM MgCl_2_, 10 mM dNTPs, and 0.5 U BIOFACT *Taq* DNA polymerase (BIOFACT, Seoul, Korea). Amplification was completed over 40 cycles.

### Isolation of peripheral blood mononuclear cells and polymorphonuclear cells

Peripheral blood mononuclear cells (PBMCs) and polymorphonuclear cells (PMNs) were isolated by the single-step centrifugation of whole blood samples onto a Polymorphprep application sheet (Axis-Shield, Oslo, Norway) according to the manufacturer’s recommendations. Blood in the EDTA tube was layered onto the application sheet at a ratio of 1:1 and centrifuged at 500×*g* for 35 min. The PMN (granulocytes) and PBMC (lymphocyte and monocyte) layers were carefully collected and resuspended in 1× phosphate-buffered saline (PBS) and then centrifuged at 14,000×*g* for 5 min to remove the supernatant. Cell pellets were stored at −80°C while awaiting RNA extraction.

### RNA extraction and cDNA synthesis

Total RNA was extracted from horse PBMCs and PMNs using TRIzol (Invitrogen, Karlsruhe, Germany) according to the manufacturer’s instructions. The purity of the extracted RNA was confirmed by measuring absorbance at 260 nm and 280 nm using a spectrophotometer (ND-1000, Nanodrop Technologies Inc., Wilmington, DE, USA). RNA of purity (optical density value of 260 nm/280 nm) greater than 1.8 was selected for further analysis and was stored at −80°C until further analysis. To synthesize cDNA, 1 μg of RNA was reverse transcribed for each sample using the SuperScript III First-Strand Synthesis System (Invitrogen, Germany), according to the manufacturer’s instructions.

### Reverse transcriptase polymerase chain reaction and real time-quantitative polymerase chain reaction

The NCBI (http://www.ncbi.nlm.nih.gov) and the Ensembl Genome Browser (www.ensembl.org) were used to retrieve gene sequence information. The primers used in the amplification of the genes (Table 1) were synthesized using the PRIMER3 software (http://bioinfo.ut.ee/primer3-0.4.0/). Reverse transcriptase (RT)-PCR and real-time qPCR were carried out using a C1000 Thermal Cycler (Bio Rad, Hercules, CA, USA) to measure the relevant expression of target genes. PCR products were separated using agarose gel electrophoresis and detected under UV light. Real-time qPCR was performed using master mixture Evagreen for 40 cycles. All measurements were carried out in triplicate for each sample, and the 2^−ΔΔCt^ method was used to determine relative gene expression. Gene expression was normalized using glyceraldehyde 3-phosphate dehydrogenase.

### Statistical analysis

Statistical analyses were performed using GraphPad Prism 6. Data are represented as the means±standard error of the mean of three or five independent samples. The results were analyzed using analysis of variance and Student’s *t*-test and were considered significant when * p<0.05, ** p<0.01 or *** p<0.001.

## RESULTS

### Changes in the blood parameters of Jeju and Thoroughbred horses after 30 min of exercise

**Figure 1:**
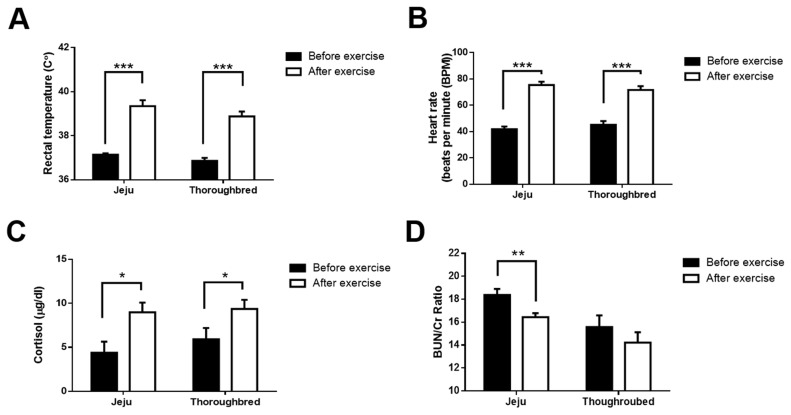
Jeju horses showing significantly increased muscle catabolism-related metabolic parameters after 30 min of exercise. Jeju and Thoroughbred horses were exercised for 30 min. Rectal temperatures were measured, and blood samples were collected for each horse both before and after exercise. (A) Rectal temperature; (B) heart rate; and (C) cortisol levels of Jeju and Thoroughbred horses before and after exercise. (D) Blood urea nitrogen (BUN) and creatinine (Cr) levels in blood samples. The BUN/Cr ratio was calculated to compare the muscle catabolism of the two breeds. Black and white circles are used to represent data from before and after exercise, respectively (n = 5 for each breed; * p<0.05, ** p<0.01, and *** p<0.001, unpaired Student’s *t*-test).

To determine whether 30 min of exercise differentially alters the blood metabolic parameters of Jeju and Thoroughbred horses, blood biochemical analyses were performed. Blood metabolite concentrations that reflect the enzymatic functions of the liver, bones and kidneys, also, immunological parameter including IgG, IgM level, WBC count and proportion of blood immune cells were not significantly affected by exercise in either of the two breeds (Table 2). However, 30 min of exercise significantly elevated rectal temperature (Figure 1A), heart rate (Figure 1B), and cortisol levels (Figure 1C) in both breeds. Although BUN and Cr levels were not significantly altered by exercise, the BUN/Cr ratio, which is negatively correlated with muscle catabolism, significantly decreased in Jeju horses but not in Thoroughbred horses (Figure 1D). Taken together, the results indicate that 30 min of exercise is sufficient to induce a stress response in horses that is characterized by increased body temperature, blood cortisol level, heart rate and muscle catabolism. We also note that the muscle catabolism-related responses of the Jeju and Thoroughbred horses differed.

### Red blood cell indices significantly increased after exercise in Thoroughbred horses relative to Jeju horses

**Figure 2:**
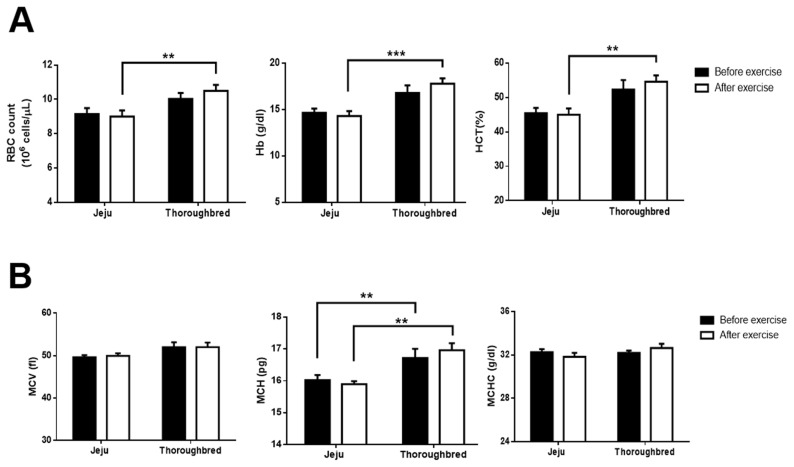
Significant increase in red blood cell indices after 30 min of exercise in Thoroughbred horses. Jeju and Thoroughbred horses were exercised for 30 min. Blood was collected before and after exercise to analyze red blood cell indices, namely, red blood cell (RBC) count, hemoglobin (Hb), hematocrit (Hct), mean corpuscular volume (MCV), mean corpuscular hemoglobin (MCH), and mean corpuscular hemoglobin concentration (MCHC). (A) RBC count, Hb, and Hct data. (B) MCV, MCH, and MCHC data. Black and white circles represent data from before and after exercise, respectively (n = 5 for each breed; * p<0.05, ** p<0.01, and *** p<0.001, unpaired Student’s *t*-test).

During exercise, the cardiovascular system increases blood flow to facilitate O_2_ transportation to muscle tissues [14]. We assessed exercise induced any changes in the RBC count, Hb level, and Hct of the blood of the two breeds in order to compare their oxygen transportation-related responses. After exercise, RBC count, Hb, and Hct tended to increase in Thoroughbred horses, resulting in Thoroughbred horses having significantly higher RBC counts and Hb and Hct levels than those in Jeju horses (Figure 2A). Next, we observed the RBC characteristics of blood samples from both breeds using light microscopy and found that RBCs of both breeds formed a rouleaux formation and were similar in size with lack of a central pallor. Then, we evaluated the RBC indices, including MCV as a marker for RBC size, MCH as an average amount of Hb per RBC, and MCHC as an indicator of the amount of Hb per unit volume. The MCV and MCHC did not differ between the two breeds. Therefore, the results from the blood parameter analysis and the blood smear assessment were consistent. Nevertheless, MCH, which represents the average amount of oxygen-carrying Hb within one RBC, was significantly higher in Thoroughbred horses than that of Jeju horses in both before and after exercise (Figure 2B). Collectively, these results suggest that Thoroughbred horses may have an increased capacity for RBC-based oxygen delivery during exercise.

### Increased neutrophil extracellular trap formation and reduced platelet count in Jeju horses relative to Thoroughbred horses following exercise

**Figure 3:**
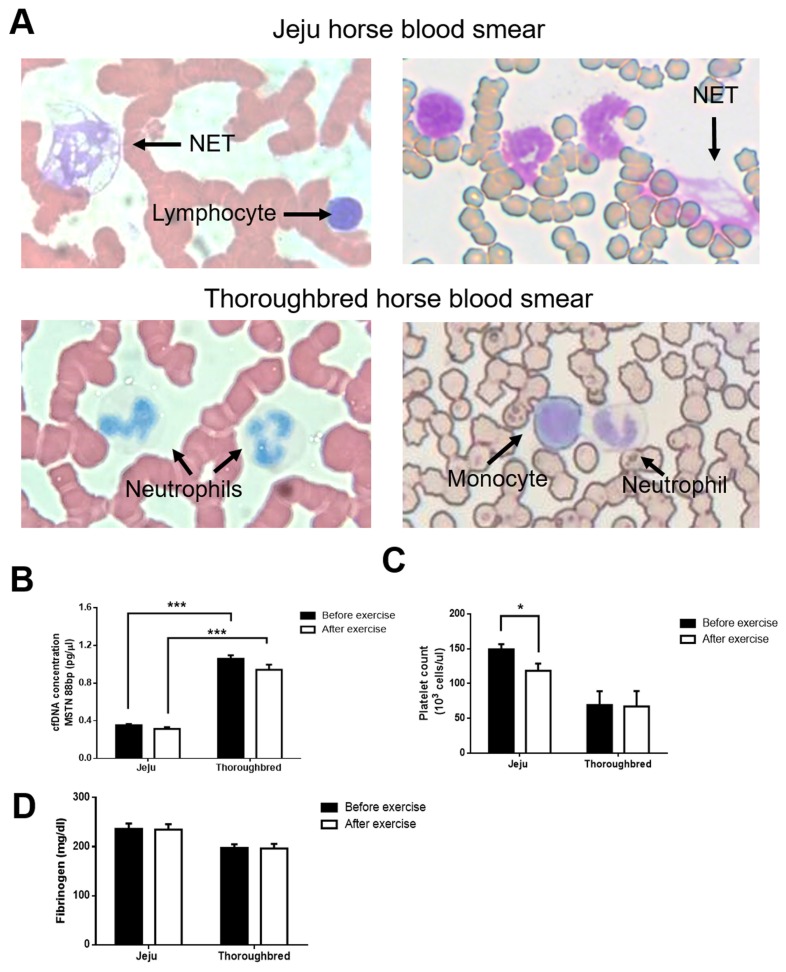
Neutrophil extracellular trap (NET) formation varies between Jeju and Thoroughbred horses, associated with platelet reduction. Jeju and Thoroughbred horses were exercised for 30 min. Blood samples were collected, and plasma was separated. (A) Blood smears from exercised Jeju and Thoroughbred horses were Giemsa stained and observed under a microscope. NETs are indicated using arrows. (B) Plasma cfDNA levels were measured using real-time polymerase chain reaction and specific primers to produce an 88 bp fragment of the chromosomal myostatin MSTN. (C) Platelet count; and (D) fibrinogen levels. Black and white circles represent data from before and after exercise, respectively (n = 5 per breed; * p<0.05, ** p<0.01, and *** p<0.001, unpaired Student’s *t*-test).

According to a study of humans, exercise can induce the release of DNA from neutrophils into extracellular space, forming neutrophil extracellular traps (NETs) [15]. NETosis, a process of NET, can be triggered by various stimuli in response to infection, tissue damage, and inflammation. In sterile inflammation, increased high mobility group box 1, citrullinated histones, and platelet-thrombin activation potentiate NETosis [16]. During NETosis, the nuclei of neutrophils swell abnormally, a process indicative of the release of decondensed chromatin and granular contents. We examined whether NETosis can be induced by exercise in horses. Blood smear samples from before and after exercise were Giemsa stained and observed under a microscope. Blood smear analysis revealed that NETosis occurred in horse blood after exercise, more so in Jeju horses (Figure 3A). Continued exercise causes tissue damage, which stimulates increases in cell free (cf) DNA in the blood [17], with one mechanism of cfDNA release being suicidal NETosis. Therefore, we measured cfDNA in the plasma both before and after exercise. Exercise did not affect cfDNA levels in either breed. However, Thoroughbred horses showed higher cfDNA levels than Jeju horses both before and after exercise. In addition to being triggered by microorganisms and tissue damage, NET formation is also triggered by platelets. NETs further stimulate platelet release and thrombosis due to their externalized chromatin, and initiate the coagulation cascade [16]. Therefore, we assessed the platelet count and fibrinogen content of blood samples, both of which are central mediators of hemostasis during coagulation. We found that platelet count was significantly reduced in Jeju horses after exercise, while platelet count in Thoroughbred horses remained unchanged (Figure 3C). Unlike platelet count, fibrinogen level was not significantly different between the breeds after exercise (Figure 3D). Therefore, NETs in Jeju horses respond more rapidly and sensitively to exercise-induced stresses than Thoroughbred horses, likely associated with differential platelet responses (cfDNA level remained unchanged and thus likely had no effect on the response of NETs).

### HSPA6 rapidly responds to exercise stress in the peripheral blood mononuclear cells of Jeju horses

**Figure 4:**
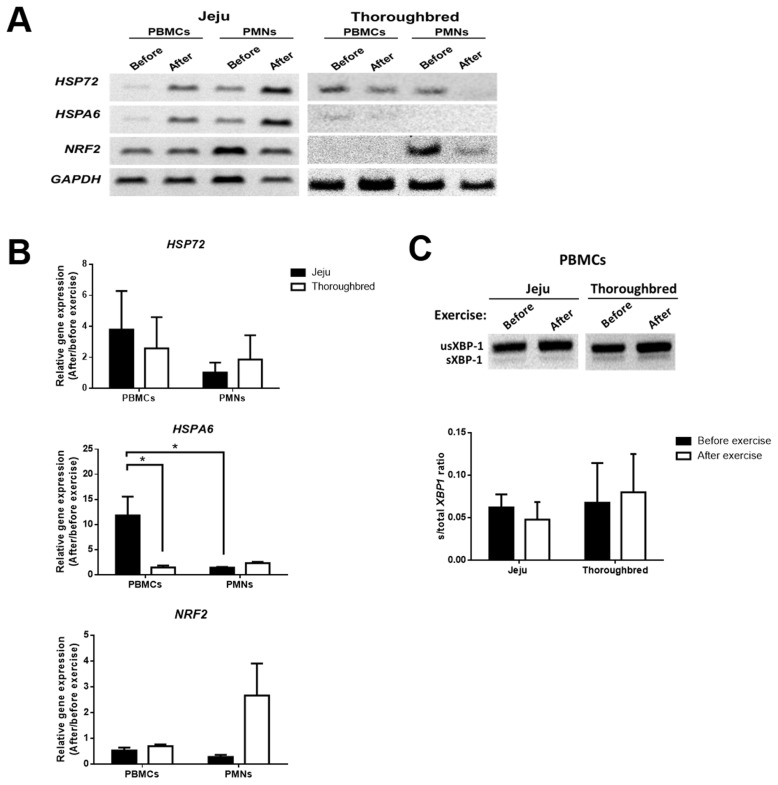
Jeju horses sensitively upregulate the stress-induced gene *HSPA6* after exercise. Jeju and Thoroughbred horses were exercised for 30 min. Blood samples were then collected. Peripheral blood mononuclear cells (PBMCs) and peripheral polymorphonuclear cells (PMNs) were separated using Polymorphprep gradient centrifugation. mRNA was extracted from PBMCs and PMNs and reversed to form cDNA to determine the expression of stress-induced genes. (A) RT-PCR of stress-induced genes. Data from one sample from each breed (Jeju and Thoroughbred) is shown. (B) Relative gene expression of stress-induced genes. Black and white bars indicate data from before and after exercise, respectively. Data is represented as means±SEM (n = 3 per breed; * p<0.05, one-way analysis of variance). (C) The expression of unsplicing (us) and splicing (s) XBP-1 was analyzed using RT-PCR and real-time PCR. The relative gene expression of splicing to total XBP-1 was calculated and plotted. Black and white bars represent data from before and after exercise, respectively. Data are represented as means±SEM (n = 3 per breed). RT-PCR, reverse transcriptase polymerase chain reaction; SEM, standard error of the mean; *HSP72*, heat shock protein 72; *HSPA6*, heat shock protein family A (Hsp70) member 6; *NRF2*, nuclear factor erythroid 2 (Nfe2)-related factor 2; *XBP-1*, X-box binding protein 1; *GAPDH*, glyceraldehyde 3-phosphate dehydrogenase.

Exercise induces endoplasmic reticulum, oxidative, and inflammatory stress in the muscle tissue [18]. Our previous study of horse muscle cells showed that heat stress increases the expression of various stress-related genes [19]. Although, it is not clear whether stresses induced by exercise can affect WBCs, neutrophils and monocytes are mainly associated with the recognition of stress-induced molecules via various receptors. As immune cell function, such as NET and platelet levels, differ between Jeju and Thoroughbred horses, their WBCs may differ in their functions and responses to exercise-induced stress. Thus, we determined whether exercise-induced stress-related gene expression took place in blood immune cells. We investigated the gene expression of stress-related genes in two populations of WBCs; PBMCs and PMNs including heat shock protein 72 (*HSP72*), heat shock protein family A (Hsp70) member 6 (*HSPA6*), and nuclear factor erythroid 2 (Nfe2)-related factor 2 (*NRF2*), which are prosurvival molecules to protect against variety of stressors. The results showed that after 30 min of exercise, *HSPA6* expression in the PBMCs of Jeju horses was higher than in Thoroughbred horses. In Jeju horses, *HSPA6* expression in PBMCs was significantly higher than in PMNs. However, the expression of *HSP72* and *NRF2* was not significantly different between the two breeds, and WBC subpopulations also did not vary significantly (Figure 4A–B). In our previous study of horse muscle cells, the ratio of the splicing form of X-box binding protein 1 (*sXBP1*) gene to total *XBP1*, which is a common indicator of endoplasmic reticulum (ER)-stress, was increased after exposure to heat-stress [19]. We investigated whether *sXBP1* in PBMCs may be affected by exercise-induced stress. However, the ratio of *sXBP1* to *XBP1* in the PBMCs in both breeds was not affected by exercise (Figure 4C). The results suggest that PBMCs and PMNs in both breeds have unique responses to exercise-induced stress. Additionally, *HSPA6* was upregulated more sensitively in Jeju horse PBMCs than in the PMBCs of Thoroughbred horses.

### CXC chemokine receptor 4 upregulation in Thoroughbred horses after exercise

**Figure 5:**
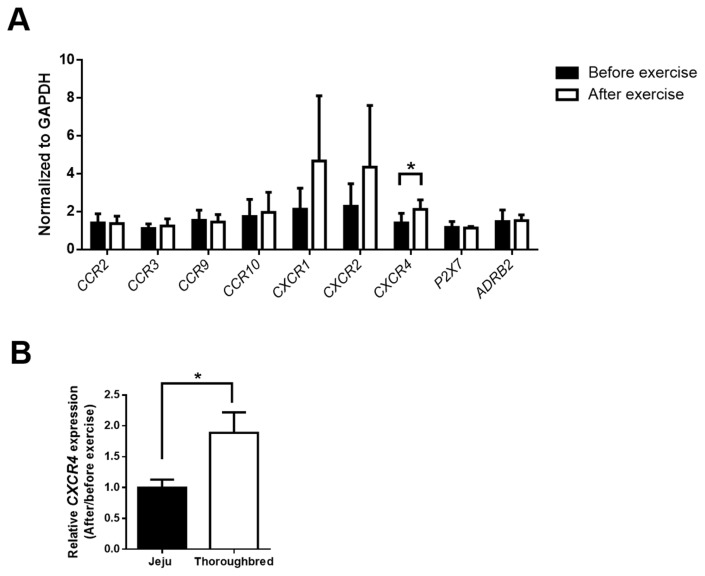
Exercise triggered the upregulation of *CXCR4* expression in the peripheral blood mononuclear cells (PBMCs) of Thoroughbred horses. Jeju and Thoroughbred horses were exercised for 30 min. Blood samples were collected and PBMCs were separated using Polymorphprep gradient centrifugation. mRNA was extracted from PBMCs and reversed to form cDNA to the determine the expression of chemokine receptor genes. (A) Results of the real-time PCR of various chemokine receptor genes. Data from Thoroughbred horses are indicated by black and white bars representing data from before and after exercise, respectively. (B) The relative *CXCR4* gene expression of the two breeds as shown by black and white bars representing data from Jeju and Thoroughbred horses, respectively. Data are presented as means±standard error of the mean (n = 5 per breed; * p<0.05, ** p<0.01, and *** p<0.001, unpaired Student’s *t*-test). *CCR*, C-C chemokine receptor; *CXCR*, CXC chemokine receptor; *ADRB2*, beta-adrenergic receptor 2.

In the blood, cytokine and chemokine receptors are mainly expressed in immune cells, and the upregulation of cytokines and chemokines involve crosstalk between immune cell functions and muscle regeneration after stress-induced tissue damage. In our previous study, interleukin 6 (*IL-6*), *IL-8*, chemokine (C-C motif) ligand 4 (*CCL4*), and chemokine (C-X-C motif) ligand 2 (*CXCL2*) expression was significantly increased after exercise in the skeletal muscles of both Thoroughbred and Jeju horses [20]. As 30 min of exercise can induce stress-related gene expression in PBMCs, we hypothesized that exercise-induced stress could alter immunogenic markers in horse lymphocytes and monocytes. Thus, we examined the expression of various genes including cytokines, chemokines, and inflammasome signaling genes (*IL-6*, *IL-8*, *IL-1B*, *CCL2*, nucleotide-binding oligomerization domain, leucine rich repeat and pyrin domain containing 3, and caspase 1). We found that exercise did not affect the expression of any of the assessed genes (data not shown). Next, we assessed whether exercise could induce changes in the expression of various chemokine receptors, including C-C chemokine receptor 2 (*CCR2*), *CCR3*, *CCR9*, *CCR10*, CXC chemokine receptor 1 (*CXCR1*), *CXCR2*, *CXCR4*, purinergic receptor P2X 7, and beta-2 adrenergic receptor in PBMCs. The results showed that the expression of only *CXCR4* was significantly altered after exercise in the PBMCs of Thoroughbred horses (Figure 5A). Furthermore, *CXCR4* expression in Thoroughbred horses was significantly higher than in Jeju horses. (Figure 5B). Overall, the upregulation of *CXCR4* after 30 min of exercise indicates that Thoroughbred horse lymphocytes may respond directly to CXCR4-ligands produced by muscle injury more rapidly than Jeju horse lymphocytes.

## DISCUSSION

The results of our study showed that a single bout of 30 min of exercise can trigger the alteration of some hematological and biochemical parameters in Jeju and Thoroughbred horses. Hyperthermia and increases in cortisol levels after exercise can induce ER stress in horses [18]. We found that muscle catabolism in Jeju horses increased after exercise. Urea is the final catabolite of endogenous protein breakdown, while creatinine is the final catabolite of muscular robust metabolism [21]. In both breeds, BUN was not altered by exercise, although in Jeju horses creatinine levels increased after exercise, leading to significant decreases in the BUN/Cr ratio. These findings suggest that the muscle tissues of Thoroughbred horses have a higher tolerance to exercise-induced muscle catabolism than that of the muscle tissue of Jeju horses.

During exercise, RBCs play a pivotal role in the trans portation of oxygen between the lungs and other tissues (oxygenation and deoxygenation) [14]. In this study, the RBC count, Hb, and Hct of the blood of Thoroughbred horses were all significantly higher than those of the blood of Jeju horses after exercise. Conversely, exercise did not affect RBC indices in Jeju horses, and the size of the RBCs of both species were unaffected by exercise. RBC and blood flow determine transportation of oxygen during exercise. In this study, we found that RBC count was higher in Thoroughbred horses but heart rate was not different from Jeju horses. Although we did not exactly examine blood flow rate in this study, these results suggests that increased RBCs numbers in Thoroughbred horses may help to promote oxygen transportation during exercise. However, it notes that there is a possibility that reduction in the volume of plasma in the blood cause increased numbers of RBC in the blood. Exercise also increased blood viscosity via dehydration caused by sweating. Prior studies have found that an increase in total Hb of 1 g per kg body weight (g/kg) can increase VO_2·max_ in athletes to greater extent than that in non-athletes [22]. This effect may explain our results. Thoroughbred horses have the best racing performance among horse breeds because they are able to quickly increase Hb-O_2_ affinity to better supply their muscle tissues with oxygen during exercise.

Acute physical exercise and its associated stresses trigger the mobilization and activation of leukocytes, platelets, and fibrinolytic pathways [23]. In our study, no changes in cfDNA levels were observed in blood of either breed after exercise, despite the induction of NETosis. cfDNA was used as an indicator of overtraining. Continuous exercise increases cfDNA levels in the blood [17], and thus, the short periods of exercise used in our study may not have been sufficiently strenuous to induce cfDNA alteration. The different physiological stresses caused by exercise can increase the amount of cfDNA in the plasma, dependent upon the intensity and duration of the exercise, the associated metabolic stress, and the inflammatory response of leukocytes [24]. cfDNA levels may not be associated with NETs during only short periods of exercise; however, NETs and reduced platelet counts were observed in blood smears from Jeju horses, which can be explained by the induction of NETosis. Activated platelets mediate NETosis via the effects of P-selectin and P-selectin glycoprotein ligand-1 during neutrophil binding. NET components such as DNA, histone, and granular proteins (elastase, cathepsin G, and myeloperoxidase) also activate platelets and serve as scaffolding for the assembly and aggregation of platelets [16]. In addition, the complement level (C4) in Jeju horses was higher than that in Thoroughbred horses (data not shown) likely because the machinery systems of NETosis and the complement and coagulation systems overlapped [25]. Relative to Thoroughbred horses, in Jeju horses, the neutrophils and platelets more actively responded to exercise-induced stresses.

Muscle degradation during exercise can activate numerous inflammatory responses. This stress-induced mechanism not only involves the muscle tissue but also the peripheral blood cells, such as the WBCs, which are important components of the immune system [26]. A single bout of exercise significantly alters human *PBMC* and *PMN* gene expression, which is characterized in many cases by the abrupt activation and deactivation of genes associated with stress, inflammation, and tissue repair [27,28]. According to the results of the stress gene expression assays in horse PBMCs and PMNs, we confirmed that *HSP72* and *HSPA6* were upregulated after exercise in both breeds. HSPs are highly conserved proteins that play key roles in cellular repair and protection. The HSP family is involved in stress resistance, protein folding, stabilization, and shuttling functions in response to stress [9]. In our experiment, *HSPA6* was clearly upregulated after exercise, more so in Jeju horse PBMCs than in Thoroughbred horse PBMCs. No significant differences in *HSPA6* expression was observed in PMNs between the two breeds. These results indicate that the lymphocyte and monocyte populations of Jeju horses can sensitively respond to exercise-induced stress even after only 30 min of exercise.

We assessed the expression of immune related gene in cluding various chemokine receptors and only *CXCR4* was upregulated following exercise. Muscle injury during exercise activated the WBCs to facilitate the alteration of the pro-inflammatory environment to an anti-inflammatory environment and aided in the regulation of the activation, expansion, and differentiation of muscle stem cells during muscle regeneration. Skeletal muscle regeneration is a complex process composed of multiple steps. Inflammatory responses play central roles in bridging initial muscle injury responses and timely muscle injury repair [29]. In human studies, PBMCs are often cultured with plasma obtained from before and after exercise or using glucocorticoid. Okutsu et al [30] found that cortisol or after-exercise plasma treatment enhanced CXCL12 augmented CXCR4 expression in T lymphocytes in a dose- and time-dependent manner [30]. Several cytokines, such as tumor necrosis factor-α, interferon-γ, and IL-1β, are secreted not only by macrophages but also by T cells to facilitate muscle regeneration [29]. CXCR4 was expressed to a higher extent in Thoroughbred horses than in Jeju horses; therefore, Thoroughbred horse immune cells may have a greater ability to mitigate muscle damaged site effectively and get involved in muscle repair process, relative to Jeju horses. Changes of chemokine expression in muscle tissue and chemokine receptor expression in blood immune cells and their functional role in muscle recovery following exercise need to be done for future study.

In this study, we identify a number of characteristics specific to Jeju and Thoroughbred horses. The differences in hematological and biochemical parameters and the variation in immunological gene expression between the two breeds may explain their unique physiological and anatomical properties. Compared to Jeju horses, Thoroughbred horse physiology is more suited to racing, as it allows for more efficient oxygen transportation, has a higher tolerance to exercise-induced stress, and lower muscle catabolism and facilitates more rapid muscle recovery. This study will aid in elucidating the linkage between exercise-induced stresses and physiological alteration. The results will prompt further studies of utilizing novel traits to improve exercise performance in Jeju and Thoroughbred horses.

## Figures and Tables

**Table 1:** Primer sets used in this study

Primer name	Primer sequence (5′ to 3′)	Tm (°C)	Product size (bp)
MSTN F	TTGGCTCAAACAGCCTGAATCC	60	88
MSTN R	TCTTGGGAAGGTTACAGCAAG		
HSP72 F	CGACCTCAACAAGAGCATCA	60	213
HSP72 R	AAGATCTGCGTCTGCTTGGT		
HSPA6 F	CGTGAGGCTGAGCAGTACAA	61	107
HSPA6 R	CCAGTTCCCTCTTCTGATGC		
NRF2 F	GAGAGCCCAGACTTCATTGC	60	173
NRF2 R	TCAACCAGCTTGTCGTTTTG		
XBP1 F	AGCTCGAATGAGTGAGCTGG	58	294
XBP1 R	ATCCATGGGGAGAGGTTCTGG		
sXBP1 F	GTCTGCTGAGTCCGCAGCAGGT	58	209
sXBP1 R	TGGGTCCTTCTGGGTAGACC		
usXBP1 F	GCAGCACTCAGTCTACGTGCG	58	275
usXBP1 R	CAGCTTGGCTGATGACGTCCC		
GAPDH F	CGCTTCCCTTCCGCACTGCT	60	229
GAPDH R	CCCGTGCTCGGCCTTGACTG		
CCR2 F	CCTTTGGGGTGATGACAAGT	62	188
CCR2 R	GCCCAAGATGCTCCTCATTA		
CCR3 F	ACTATGTTTGGCGGGATGAG	62	201
CCR3 R	CCCAGGTGAAGACACTGGTT		
CCR9 F	CAGACCTGGAGGCAGAAAAG	62	237
CCR9 R	GCAGCAAGCCATGACTACAA		
CCR10 F	CCATCTCTGGCCTCTACTCG	62	200
CCR10 R	CTGGCTGAAAAGGAGAGCAG		
CXCR1 F	CTTCCGGGACATTTGAAAGA	62	212
CXCR1 R	AGTCAGAACGGGGTGATACG		
CXCR2 F	ATGCCCTGGTCGTCATCTAC	62	154
CXCR2 R	GTCAAGGCAAAGAGCAGGTC		
CXCR4 F	CAGCAGCAGGTAGCAAAGTG	62	189
CXCR4 R	TTGAAATGGGCATTCTCCTC		
P2X7 F	TCTTTGGGATCCGTTTTGAC	62	191
P2X7 R	TTCGCAGTACTTGCAACAGG		
ADRB2 F	AAATGTGGACTTTCGGCAAC	62	195
ADRB2 R	CTGACACGATCCACACCATC		

MSTN, myostatin; HSP72, heat shock protein 72; HSPA6, heat shock protein family A (Hsp70) member 6; XBP1, X-box binding protein 1; sXBP1, splicing form of X-box binding protein 1; usXBP1, un-spliced X-box binding protein 1; GAPDH, glyceraldehyde 3-phosphate dehydrogenase; CCR, C-C chemokine receptor; CXCR, CXC chemokine receptor; P2X7, purinergic receptor P2X 7; ADRB2, beta-2 adrenergic receptor.

**Table 2:** Biochemical analysis, immunological analysis, white blood cell count and proportion of blood immune cells of Jeju and Thoroughbred horses before and after 30 minute exercise

Parameters	Jeju (n = 5)		Thoroughbred (n = 5)	
	
	Before exercise	After exercise	Before exercise	After exercise
Aspartate aminotransferase (U/L)	295.00±17.62	298.20±20.68	258.60±14.31	268.60±13.45
Alanine aminotransferase (U/L)	6.40±0.51	5.80±0.58	8.00±1.27	8.60±1.69
Lactate dehydrogenase (U/L)	459.20±22.71	470.20±41.53	399.00±27.15	408.80±25.39
Creatine kinase (U/L)	470.00±35.25	435.20±76.24	396.00±80.85	425.60±75.99
Alkaline phosphatase (U/L)	138.20±13.03	141.00±16.74	158.60±10.18	159.00±9.21
Total bilirubin (mg/dL)	1.06±0.31	1.10±0.35	1.56±0.31	1.68±0.07
Blood urea nitrogen (mg/dL)	20.40±0.87	20.20±0.97	19.40±1.00	19.20±1.16
Creatinine (Cr, mg/dL)	1.11±0.06	1.23±0.08	1.23±0.08	1.36±0.08
Immunoglobulin G (mg/dL)	456.40±15.13	463.20±16.00	421.80±23.02	419.40±20.37
Immunoglobulin M (mg/dL)	60.80±12.39	58.80±11.22	114.60±21.66	112.40±20.46
White blood cell count (10^3^ cells/mL)	12.39±0.76	12.95±0.82	8.69±0.52	9.34±0.75
% Lymphocytes	57.54±2.99	61.58±2.98	36.80±3.14	33.24±3.07
% Neutrophils	36.80±3.14	33.24±3.07	52.26±1.62	51.54±2.04
% Monocytes	4.38±0.65	3.94±0.46	4.02±0.66	3.26±0.24
% Eosinophils	0.54±0.32	0.68±0.49	1.16±0.73	1.18±0.75
% Basophils	0.74±0.17	0.56±0.09	0.46±0.05	0.52±0.08

Data represented mean±standard error of the mean (n = 5 per breed).
